# The intuition of neutrality and consequentialist thinking: potential antinatalist implications

**DOI:** 10.1186/2193-1801-2-99

**Published:** 2013-03-11

**Authors:** Karl Pettersson

**Affiliations:** Department of Philosophy, Uppsala University, Box 627, SE-751 26 Uppsala, Sweden

**Keywords:** Intuition of neutrality, Population ethics, Necessitarianism, Antinatalism, Consequentialism

## Abstract

Many people seem to share some version of what has been called the “intuition of neutrality” about creating new people, which, roughly, says that there exists a certain range of levels of well-being such that creating people within this range is, in itself, morally neutral, but creating people with a level of well-being outside this range is not morally neutral. In this paper, I will discuss different interpretations of this intuition, and specifically distinguish between what I will call counterfactual interpretations and Do-interpretations of the intuition. I will argue that it is hard to interpret the intuition in a way that does not give rise to antinatalist moral reasons, i.e. reasons favoring an empty future population, when it comes to choices of social policy. In particular, this holds if we assume a conception of relevant outcomes of actions reflecting consequentialist moral intuitions. In the end, I will formulate a normative principle of welfare promotion which I argue respects the most plausible counterfactual version of the neutrality intuition.

## Basic aims, assumptions and notation

The aim in the first part of the paper, up to section ‘A Welfarist principle respecting a (NeuCf)-like intuition of neutrality’, where the different interpretations of the neutrality intuition are discussed, is mainly descriptive. In this part, I will point to a potential conflict within one common sense conception of morality. In this context, a common sense conception of morality may be looked upon as a set of moral norms that has gained widespread acceptance within modern societies. In determining the content of common sense conceptions of morality, I will mostly appeal to moral intuitions recognized as familiar from modern philosophical literature. However, I do not claim that the conception of morality that gives rise to the problem is shared by all, or most, people in the current world, or even some current society. In section ‘The intuition’, I make a taxonomy of different versions of the neutrality intuition, with differing boundaries of the range of welfare levels, such that bringing about people at these levels is ethically neutral, with respect to the welfare of the potential people. In section ‘The counterfactual interpretations’, I describe *counterfactual* interpretations of the neutrality intuition, where the bringing about is interpreted in counterfactual terms: you bring about people by performing an action just in case the existence of those people is counterfactually dependent of the performance of that action. In section ‘The counterfactual interpretations and antinatalism’, I discuss some antinatalist (i.e. giving moral reasons against creating new people) implications of these interpretations. In section ‘The do-interpretations’, I describe a new class of *Doing*-interpretations, and discuss their relation to the counterfactual interpretations, and I discuss potential antinatalist implications of these interpretations. In that section, I also discuss *pro tanto* implications of these interpretations for policy choices.

I leave it open whether we should accept the antinatalist *pro tanto* reasons yielded by different versions of the intuition, and I also leave it much open to what extent they may be outweighed by other reasons, making it all things considered permissible to create new people. In section ‘A Welfarist principle respecting a (NeuCf)-like intuition of neutrality’, I formulate a principle of welfare promotion, which may be looked upon as a criterion of rightness, if you are a Utilitarian, or (with an added *ceteris paribus* clause) as a formulation of the proper subset of morality concerned with promoting welfare, if you are a deontologist, or a consequentialist who acknowledges morally relevant final values other than welfare. This principle is meant to appeal to those who find the (NeuCa)-version of the intuition appealing, and it has the *pro tanto* antinatalist implications of this version of the intuition. In an appendix, I give a more formal treatment of the different interpretations of the neutrality intuition.

I assume that levels of well-being can be represented by real numbers (and that there are thus no incommensurabilities of well-being). In the examples, I assume that there are non-empty sets of possible individuals that would exist, and that there are true answers to the question what their level of well-being would be, given that certain actions are performed. In reality, it might not always be true that a certain individual *x* would exist, or would not exist, if a certain action were performed, or more general, if a certain proposition *p* were true (it might be that *x* merely *might* exist if *p* were true). However, various sorts of counterfactual indeterminacy would spell trouble for most normative principles concerned with actual outcomes (cf. (Carlson 1995) and (Horty 2001)), and I make no attempt to solve these problems in this paper.

The quantifiers ∀ and ∃ should be read as ranging over all possible individuals. We also assume a existence predicate, E, such that E(*x*) denotes a statement saying that the possible individual *x* actually exists^a^.

Let propositions of the form *lw*(*x*) = *a* state that the well-being of individual *x* equals *a*. Let *pq* denote conditional statements, with a counterfactual operator, in the style of David Lewis (Lewis 2001). Thus, *plw*(*x*) = *a* means that if *p* were the case, *x*’s level of well-being would be *a*. I assume that the bearers of deontic status are states of affairs, and I will in this context focus on statements of the form Do(*x*,*p*), in the style of Stig Kanger (Kanger 2001[1972]), read that *x* sees to it that *p* (and negated statements of this form, i.e. statements of the form ¬Do(*x*,*p*)). I assume that reasons against seeing to it that *p* for *x* are to be interpreted as reasons for ¬Do(*x*,*p*) rather than Do(*x*,¬*p*). If we have moral reasons against seeing to it that a certain state of affairs is the case, we need not have moral reasons for seeing to it that its negation is the case.

The level of well-being for *x* may be some aggregate sum of some function of the hedonic levels, satisfied or dissatisfied preferences or achievements of some items on an “objective list” during the different parts of the life of *x*: I will not discuss substantial axiological questions here. Nor will I discuss identity criteria for individuals, even though a full moral theory where the distinction between contingent and necessary individuals plays a fundamental role (which is the case with the principle I propose in section ‘A Welfarist principle respecting a (NeuCf)-like intuition of neutrality’) would have to answer these questions e.g. in order to assess the moral status of abortions.

## The intuition

In *Weighing Lives*, John Broome describes what he calls an “intuition of neutrality” about the addition of people to the world (Broome 2004, p. 143–145). He describes a couple wondering whether to have a child, and ending up with the decision to remain childless, because they think their own lives will be better on balance without children. Many people tend to think they are not acting wrongly, and that there is not even a slight moral reason to have a child, stemming from the child’s potential well-being, that is outweighed by the couple’s benefits from childlessness. According to the intuition, there is some range of levels of well-being such that bringing about people living their lives at those levels is, in itself, morally neutral.

However, few people think that there never can be any reasons for or against having children, related to the children’s well-being. For example, many would claim that there are strong moral reasons against having children if these children would live their lives at a very low level of well-being. Broome points out that there may, or may not, be an upper limit, as a well as a lower limit, to this neutral range. If there is an upper limit, we have moral reasons to add people with a level of well-being above that limit, for the sake of the well-being of these people. Let us distinguish between some types of neutrality intuitions that differ from each other as to how they specify the range of neutral levels (where *a* and *b* are reals): ***(Unbounded)*** The range is (−*∞*,*∞*). This type of neutrality intuition will not be discussed any further. I think it has poor support in common sense, because it implies that the fact that our potential children would live at the lowest level of welfare possible cannot be counted as a moral reason against having children. ***(Upper bound only)*** A range of the type (−*∞*,*b*]. This type will not be discussed any further, for the same reasons as the former type. ***(Lower bound only)*** A range of the type [*a*,*∞*). Neutrality with a lower, but no upper, bound. This type of neutrality can be regarded as equivalent with what has been called *The Asymmetry* (Arrhenius 2000, p. 137)i.e. that we have moral reasons speaking against creating children that would have poor lives, but no moral reasons *per se* in favor of creating children with good lives. This type of neutrality intuition is the basis of the interpretations (NeuCf) and (NeuCa), which I discuss in sections ‘The counterfactual interpretations’ and ‘The do-interpretations’. ***(Lower and upper bound)*** A range of the type [*a*,*b*]. This type of neutrality intuition is the basis of the interpretations (NeuCfUB) and (NeuCaUB), which I discuss in sections ‘The counterfactual interpretations’ and ‘The do-interpretations’. Note that, if we used a degenerate interval, like [*a*,*a*], we would essentially be denying the intuition of neutrality.

The intuition may, as in the recapitulation above, be stated directly in deontic terms, i.e. in terms of moral reasons or obligations, or it may be stated axiologically, i.e. in terms of the value of outcomes, populations or other value-bearers. Broome himself considers different axiological interpretations, because he is primarily interested in questions about value, which he ultimately rejects, but he considers the possibility of interpreting the intuition in deontic terms (Broome 2004, p. 148).

## The counterfactual interpretations

It might appear natural to interpret the limited intuition of neutrality counterfactually, so that any individuals whose existence is counterfactually dependent on the performance of a certain action might give rise to moral reasons for or against that action, in terms of their own potential welfare, if their welfare would lie outside a certain neutral range. Thus, we might consider a pair of counterfactual interpretations of the intuition.

(NeuCf) states that if a certain individual *x* would exist if a proposition of the type Do(*y*,*p*) were true, but not if that statement were not true, there is a moral reason against Do(*y*,*p*), in terms of the welfare of *x*, if *x* would live her life below a certain lower bound of the neutral range *a*, but there is no reason for Do(*y*,*p*), in terms of the welfare of *x*, if *x* would live her life above *a*.

(NeuCfUB) is like (NeuCf) except for the assumption that there is a closed, proper interval of neutral levels, with both a lower and an upper bound, we can interpret the intuition as follows. Thus, (NeuCfUB) adds to (NeuCf) that there is a moral reason for Do(*y*,*p*) in terms of the welfare of *x*, whose existence is counterfactually dependent on Do(*y*,*p*), if *x* would live her life above the upper bound of the neutral range. For a more formal treatment of (NeuCf) and (NeuCfUB), see ‘The counterfactual interpretations’.

As stated, (NeuCf) and (NeuCfUB) cannot, of course, in themselves give any substantial normative guidance, because we have not specified the *a* and *b*, i.e. lower and upper bounds of the neutral range. For present purposes, it is unnecessary to do so. The lower bound of the neutral range, *a*, may, or may not, be identical with a “zero level” of well-being, such that living is just as good as not living for a person living at that level. If we want to capture some intuition that is widely shared people by living in the present world, we cannot place the lower bound of the neutral range below the zero level, I think.

## The counterfactual interpretations and antinatalism

Let *EF* denote the collective consisting of the couple Eve and Frank, wondering about whether to have a child. Let *c* stand for the statement that they conceive a child at a certain time. Assume that if they were to see to it that *c*, their child Doris, denoted *d*, would exist and live her life at a level of well-being *a*+ within the neutral range [*a*,*b*]. So, Do(*EF*,*c*) *lw*(*d*) > *a*∧Do(*EF*,*c*) *lw*(*d*) < *b* is true, and there is thus no moral reason for Do(*EF*,*c*), rather than its negation, or vice versa, in terms of the well-being of the conceived child, Doris. However, there may be, of course, reasons for conceiving or not conceiving, in terms of other considerations, e.g. the positive and negative effects of the existence of Doris on the welfare of other people. I want to focus on a certain class of considerations. It is probable that Doris, if she is brought into existence, eventually will have children and grandchildren, and so on.

Let *CB* denote the set of individuals “brought about” by Eve’s and Frank’s conceiving, in the sense that they would exist just in case Eve and Frank were to conceive (see ‘Sets of individuals’ for exact definition). *CB* will probably contain many individuals. Assume that some individuals brought into existence, live their lives below *a*, the lower bound of the neutral range, which is plausible, I think, for any reasonable choice of *a*. Then, there is a risk that ∃*x*[Do(*EF*,*c*) *lw*(*x*) < *a*∧¬Do(*EF*,*c*) ¬E(*x*)] is true, i.e. some individuals whose existence would be counterfactually dependent on Eve’s and Frank’s conceiving would live their lives below the lower bound *a*, and there is thus a moral reason against Do(*EF*,*c*) according to (NeuCf) and (NeuCfUB). If reasons of this type are not outweighed by positive moral reasons for conceiving, it seems that the intuition of neutrality, if we accept the counterfactual interpretations, in practice tends towards antinatalism, i.e. the view that conceiving children is generally morally wrong. In recent times, the antinatalist view has been defended by e.g. David Benatar (Benatar 2006). Benatar’s main argument is that the existence of bad things in the life of any individual implies that the individual is harmed by being brought into existence, while the existence of good things does not imply that the individual is benefited by being brought into existence.

Is it plausible to claim that these negative reasons are, generally, outweighed^b^? If we assume (NeuCf), we cannot, as the principle is defined, say that the reasons against Do(*EF*,*c*) derived from the negative well-being of people in *CB* below the *a*-level are outweighed by positive reasons derived from the well-being of people in *CB* living above the *a*-level, because the existence of people living good lives does not give rise to moral reasons in terms of the well-being of those people, according to (NeuCf). However, there might be other reasons for creating new people, and I discuss that below. If we assume (NeuCfUB), we might claim that the well-being of people in *CB* living above the upper bound, *b*, gives rise to reasons for Do(*EF*,*c*), if *b* is not placed very high. However, I am inclined to consider (NeuCfUB) less plausible than (NeuCf), as an interpretation of any widespread intuition of neutrality. If the point of the neutrality intuition is that, while you harm a person by creating if she would live an unhappy life, you do not benefit a person, in any morally relevant sense, by creating her in the case where she would live a happy life, this must also apply to cases where she would live an extremely, as well as a moderately, happy life. We can compare with Jan Österberg, who writes that “[m]orality …is concerned with the weal and woe of sentinent beings …[a]nd you are not concerned with the weal and woe of a person if you think it is better that he exists and is happy than that he does not exist’, but “you are thus concerned if you think it is better that he does not exist than that he would have an unhappy life” (Österberg 1996, p. 97).

However, it also seems to be a common sense view that we may have moral reasons (related to well-being) to prefer one outcome to another even in some cases where the first outcome is not better for any individual existing in both outcomes. I am thinking of cases like the so-called Non-Identity Problems, described by Derek Parfit in *Reasons and Persons* (Parfit 1984, p. 357–363). In these cases, we have to choose between outcomes with different sets of existing people, and it seems plausible that we have moral reasons to prefer the outcome *W* to an outcome *W*^′^, if the level of well-being in *W* (for the individuals existing in *W*) is higher than the level of well-being in *W*^′^ (for the individuals existing in *W*^′^)^c^. Could this help us in avoiding the anitnatalist tendency of (NeuCf)?

Return to Eve and Frank, and their potential conceiving.

Let *NCB* denote the set of individuals that would exist if Eve and Frank were not to conceive Doris, but not if they were to conceive Doris. The intersection *CB*∩*NCB* is always empty. It seems realistic to think that *NCB* is non-empty. If Doris were never to exist, the person with whom she eventually would have had children if she had existed, might then find another partner, and conceive children with different identities. It might be that *NCB*, if Eve and Frank were not to conceive, would contain more people than would *CB*, if they were to conceive, living under the lower bound, *a*, of the neutral range, or that any person in *NCB* would live at a lover level of well-being than any person in *CB*. Then, we have different types of Non-Identity Cases, and it seems plausible to claim that there are moral reasons for Eve and Frank to conceive. But it seems that Eve and Frank cannot in any simple way appeal to the neutrality intuition, in order to claim that conceiving is something morally optional: they have significant moral reasons against conceiving, which may, or may not, be outweighed by considerations about the well-being of e.g. people that would exist if they were not to conceive.

## The do-interpretations

An alternative strategy would be to interpret intuition of neutrality in terms of what is done, so that the well-being of a possible individual might give rise to moral reasons for or against performing an action consisting in the bringing about of the existence of that individual, as opposed to reasons stemming merely from the fact that an individual would exist given that a certain action was performed. The potential differences between these interpretations are discussed below.

As the counterfactual interpretation, the doing-interpretation might be specified without an upper bound of the neutral range. Let (NeuCa) and (NeuCaUB) denote the doing-interpretation without and with an upper bound. With these interpretation, the counterfactual dependencies in (NeuCf) and (NeuCfUB) are replaced with references to the existence of individuals and their welfare in the scope of the Do-operators. For a more exact formulation of these principles, see ‘The do-interpretations’.

Are the pairs (NeuCf)/(NeuCa) and (NeuCfUB)/(NeuCaUB) equivalent in the sense that they, given the same choice of *a* (and *b*, for the upper-bound variants), yield moral reasons for and against the same states? They need not be if we use a standard interpretation of the Do-operator, like the one proposed by Stig Kanger (Kanger 2001[1972]). We only have to look at the so-called sufficient condition aspect of agency to ensure that this is not the case. Kanger uses the notation Dó (*x*,*p*) to express that something *x* does is sufficient for *p*, which is implied by Do(*x*,*p*). (Dó (*x*,*p*) is true at a world *w* just in case *p* is true at every world *W*^′^ that stands in a certain relation to *w*, *Sds*(*w*,*w*^′^). The intuitive interpretation of *Sds*(*w*,*w*^′^) is that *x* performs the same actions in *W*^′^ as in *w*. Even if it were the case that a certain proposition *q*, would be true at the nearest possible Do(*x*,*p*)-world in relation to a world *w*, *f*(*w*,*p*), so that the counterfactual Do(*x*,*p*) *q* is true at *w*, Do(*x*,*q*) need not be true at that world (or any world possible relative to *w*), because *q* might be false at some world *W*^′^, such that *Sds*(*f*(*w*,*p*),*w*^′^).

Thus, in the example with Eve and Frank, it might be true both that if they were to conceive Doris, some individual, *i* would come into existence, and that *i* would not come into existence otherwise, but Do(*EF*,*c*) might still not imply Do(*EF*,E(*i*)). Is it also philosophically plausible to claim that we in many cases do not bring about the existence of individuals who would not have existed if we had acted otherwise than we did? In his monography *Consequentialism Reconsidered* (Carlson 1995), Erik Carlson discusses the problem of finding a notion of action outcomes relevant for consequentialism. According to the simple notion WO, the relevant outcome of an action is just the total state of affairs that would be actual if the action were performed, and according to the notion FO, the relevant outcome is the total future state of the world from the beginning of the action (Carlson 1995, p. 10.) But Carlson also discusses different interpretations of the notion of the relevant outcome as the “causal consequences” of actions (CO1–CO10), defined in terms of counterfactual dependence of outcomes on actions. Some of the CO-notions are axiologically equivalent to WO and FO, i.e. the consequentialist moralities they would support, would contain the same set of obligations. Carlson notes that these versions “allow state of affairs that we would not normally call ‘consequences’ or ‘effects’ of a certain action, to be parts of the outcome of this action’ (Carlson 1995, pp. 55). We would usually “not call a state of affairs *s* a ‘consequence’ …of agent *P*’s action *a* if the causal path from *a* to *s* is too long and complicated”, especially if this “path involves many other *actions*, on the part of *P* or other agents” (Carlson 1995, p. 55).

Carlson claims that CO-notions not axiologically equivalent to WO or FO should not be used in the formulation of a consequentailist morality, because they fail to capture “the fundamental idea of conequentialism”, that morality is all about “making the world as good as possible” (Carlson 1995, p. 56). I am inclined to agree with this, but I also think the observation that certain counterfactual dependencies between an action and a state may disqualify the state from being regarded a consequence of an action should be taken seriously, if we want to analyze a notion of “bringing about” inherent in an intuition belonging to common sense morality.

Thus, it seems that if we interpret the intuition of neutrality along the lines of (NeuCa) or (NeuCaUB), we can in a plausible way avoid the antinatalist tendencies of (NeuCf) in typical cases where we consider an individual couple’s decision about whether or not to have a child. It might be that Eve and Frank, if they conceive Doris, only bring about the existence of Doris, and not of the other people in the set *CB*, because their existence would be counterfactually dependent on many other actions besides the conceiving of Doris, and would thus not be a consequence, in the relevant sense, of their conceiving. Of these two interpretations, (NeuCa) might be more plausible than (NeuCaUB), for the reasons given for (NeuCf) in favor of (NeuCfUB).

## The neutrality intuition and policy choices

Derek Parfit discusses cases where the identity of future people depends on choices at the political level, like his famous Depletion case, where we have to choose between different policies with lesser or greater conservation of resources, and we, in a couple of centuries will have a situation with no living person whose existence is not dependent on the choice of policy (Parfit 1984, p. 360–363)^d^. The counterfactual chains between the policy choice and the identity of the future people will in many cases be rather complex; the identities will depend on such things as people’s choices about when, and with whom, they will have children. Nevertheless, it seems that, according to common sense morality, the existence of the different groups of people is included in the morally relevant consequences of the different policy choices. The notion of morally relevant common sense-morality consequences thus seems to be sensitive to the type of choice we are considering, and this will have important consequences for the implications of the neutrality intuition when we consider choices of social policies. Consider the choice between the following alternatives. ***(Sterility)*** As a result of some kind of disease, *D*, human fertility will decline, so that the number of new people will decline, and humanity will cease to exist in a couple of generations. ***(Treatment)*** As a result of international co-operation, scientists find a cure for *D*, and humanity continues to exist for many thousands of years, with an average level of well-being well above the lower bound of the neutrality range.

What could the moral reasons for or against the cure-finding in (Treatment) be, as regards the implications of (NeuCf) and (NeuCa)? Let *I* denote a collective of international decision-makers, researchers and so on, and *t* denote the proposition that a treatment for *D* is found and implemented. Consider first Do(*I*,*t*) under (NeuCf). We have the set *TB*, of large cardinality, containing the people that will exist under *t*, and would not exist under ¬Do(*I*,*t*), where the scenario (Sterility) would be realized. Most of the *TB*-people will live their lives with a level of well-being above the neutral level, but some of them will live below that level, and the existence of *TB*-people below the neutral level gives us moral reasons for ¬Do(*I*,*t*) rather than Do(*I*,*t*), according to (NeuCf), as in the case with Eve and Frank.

What about (NeuCa)? Consider a case with an institution giving fertility treatments, where it turns out that a very large fraction of the children born as a result of their parents using this treatment will live their lives with so severe injuries that they fall under the neutral level, as a result of some unforeseen side-effect of the treatment, which, however, could have been anticipated if more careful testing had been done. According to common sense, the institution could be held responsible for these effects. It is plausible, I think, to claim, that an agent *x* only can be repsonsible for effect brought about by *x*. If so, the institution must be said to have brought about the existence of the unfortunate people. Why should not then *I* be said to have brought about the existence of *TB*-people living below the neutral level? If so, even (NeuCa) yields moral reasons for ¬Do(*I*,*t*) rather than Do(*I*,*t*), which cannot be outweighed by moral reasons for Do(*I*,*t*), in terms of the well-being of the *TB*-people living above the neutral level. However, it seems unlikely that common sense would give an antinatalist all-things-considered verdict, preferring (Sterility) in this case. Then, if common sense morality is not simply incoherent, there must be other considerations inherent in common sense morality that make it permissible to prefer (Treatment). Investigating into these reasons is beyond the scope of this paper. However, it seems possible that there are e.g. some considerations in common sense morality that permit giving greater weight to the interests of present people, or people living in the near future, and that these considerations then may give rise to moral reasons outweighing the reasons for ¬Do(*I*,*t*) rather than Do(*I*,*t*) given by (NeuCf) or (NeuCa).

One alternative type of strategy, would consist in denying that common sense morality contains a neutrality intuition in any of the senses discussed above, and only claim that it contains something like an intuition about what Gustaf Arrhenius has called *Weak Asymmetry*. According to Weak Asymmetry, we indeed might have moral reasons for creating individuals with positive welfare stemming from the welfare of these individuals, but these moral reasons “can always be overridden by some other consideration such as, for example, parental autonomy)” (Gustaf Arrhenius, Population ethics, fortchoming). Thus, the reasons would not give rise to a moral requirement to create new individuals against the wishes of potential parents. If we accept this, some of the claims stated in section ‘The intuition’, e.g. “that there is not even a slight moral reason to have a child, stemming from the child’s potential well-being”, obviously cannot be taken at face value. A further assessment of this strategy is beyond the scope of this essay.

## A Welfarist principle respecting a (NeuCf)-like intuition of neutrality

In this section, I postulate the normative principle (NeuW), which I think is welfarist in spirit, aimed to take care of a neutrality intuition without upper bound for the neutral level, and with the lower bound set at the zero level of welfare, i.e. the level such that life is not worth living for an individual living below that level. For example, creating people with positive welfare “for their own sake” is not required, according to the principle, and it is not permitted to create people with positive welfare at the cost of decreased welfare of people existing no matter what we do (necessary people), and it is not permitted to create people with negative welfare, if this can be avoided, and is not outweighed by benefits for existing people. The relevant outcomes are whole worlds, which, as argued in section ‘The do-interpretations’, I think is in accordance with the intuitions behind consequentialist ethics. However, I do not define the principle in terms of any explicit value-ranking of outcomes. Such a ranking would obviously have to be relativized to a particular choice set, relative to which people may be necessary or contingent.

In the following discussion, let us assume that we have a non-empty set of worlds *W* that are accessible in a given context of choice. Here, it is not necessary to deal with multiple contexts (e.g. choice situations facing different agents, or the same agent at different times), even though a full elucidation of the deontic concepts would have to do that. Let *I*_*N*_ denote the set of necessary individuals, i.e. the individuals existing in all worlds in *W*, *I*_*C*_(*w*) the set of contingent individuals relative to a world *w*, i.e. the set of all individuals existing in *w* but not in all worlds in *W*, and *tw*(*I*) the total welfare of the individuals in set *I*. If *I* is empty, let *tw*(*I*) = 0.

In accordance with what is stated in section ‘Basic aims, assumptions and notation’, I do not intend the principle to handle cases with indeterminacy as to whether some individuals would exist at certain levels of well-being, given certain courses of action. Let us then assume that for any proposition *p* (or more specifically, an action-proposition of the type Do(*x*,*q*)) that is regarded as possible in the context, a determinate world *w*∈*W* would be actual if *p* were true. ***(NeuW)*** Do(*x*,*p*) is permitted iff there is a world *w*∈*W* such that *w* satisfies the following conditions with respect to any subset *W*^′^ of *W* which includes *w*:

1. The total welfare of the *W*^′^-necessary individuals (i.e. the individuals existing in all worlds *w*^′^∈*W*^′^) in *w* is at least as high as the welfare of these individuals in any other world in *W*^′^, and *w* contains no individuals that are *W*^′^-contingent (i.e. not *W*^′^-necessary), OR
2. The total welfare of the *W*^′^-necessary individuals in *w* is at least as high as the welfare of these individuals in any other world in *W*^′^, and the total welfare of the contingent individuals in *w* is also at least as high as the welfare of the contingent individuals in any other world in *W*^′^ and all *W*^′^-contingent individuals in *w* have a total welfare above the zero level OR
3. if there is no world satisfying any of the above conditions, *w* is included in a subset *W*^′′^ of *W*^′^, consisting of all the worlds in *W*^′^ where all *W*^′^-contingent individuals have a total welfare above the zero level and the total welfare of all individuals in *w* is at least as high as the total welfare of all individuals in any other world in *W*^′′^ OR
4. if there is no world satisfying any of the above conditions, the total welfare of all individuals in *w* is at least as high as the total welfare of all individuals in any other world in *W*^′^.

**Figure 1:**
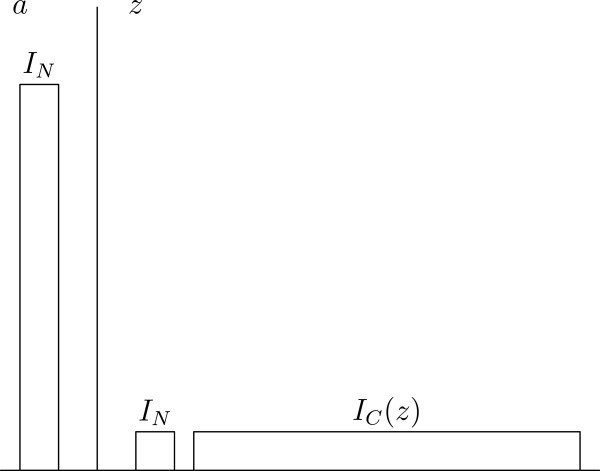
**The Repugnant Conclusion.**
  where *a*0 is empty, *I*_*C*_(*a*)⊂*I*_*C*_(*z*), and *I*_*C*_(*a*) = 1 at *z*. In this case, *a*0, and *a* are the only permissible worlds (see Figure 2). There is an overlap of individuals between *a* and *z*, but not between any other pair of worlds, and with respect to the smaller set *W*^′^−={*a*,*z*}, *a* is the only world satisfying condition 1 or 2 (remember that the individuals in *a* are necessary relative to the set *W*^′^−). However, assume that we have the set of worlds

This principle does not lead to normative dilemmas, i.e. situations where no world is permissible, because it is easy to see that for any non-empty set of worlds, at least one world satisfies at least the fourth condition, provided that there are no infinite series of better and better worlds^e^.

(NeuW) has the following implications in relation to the main problem discussed in this paper, and some other areas which have been central to the debate about population ethics during the last decades (for a survey of this debate, see (Arrhenius et al. 2010).):

1. It is always permitted to omit adding any contingent people, if adding would confer no benefit to the necessarily existing people in a set of alternative worlds.
2. It is not permitted to add contingent people, if the only optimal state of affairs as regards the welfare of the necessary people, is a state with no contingent people. In particular, we avoid some versions of Parfit’s so-called Repugnant Conclusion (implied by e.g. Classical Utilitarianism) (Parfit 1984, p. 387–391), where a large population with each individual living at a very low level of welfare is substituted for a small population with each individual living at a very high level of welfare. Assume that we have the set of worlds
  Here, *a* is the only permissible world, because it is the only world satisfying condition 1 or 2 (see Figure 1). Assume that we have the set of worlds

**Figure 2:**
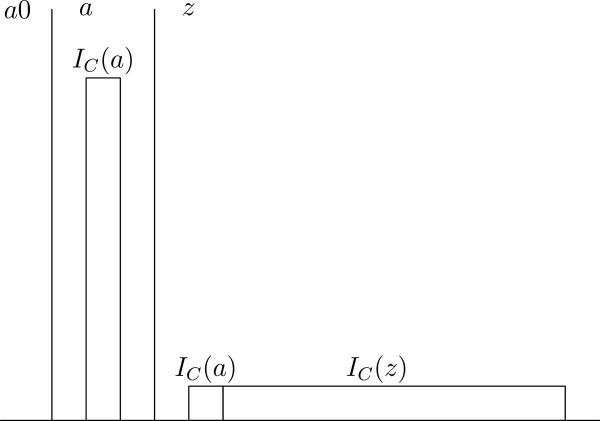
**The Repugnant Conclusion with one possible empty world.**
  where *a* contains 1000 individuals living at level 50, and *z* contains 10^6^ individuals living at level 1, and there is no overlap of individuals between *a* and *z*, *a*0 and *z*, but not *a*, are both permissible (see Figure 3). This is because *a*0 trivially satisfies condition 1, and *z* satisfies condition 2, but *a* satisfies neither condition Because of this Utilitarian aggregation in such cases, there are situations where (NeuW) violates Gustaf Arrhenius’s so-called Normative Quality Condition (Arrhenius 2000, p. 191f), and Weak Quality Addition Condition (Arrhenius 2000, p. 194f), regardless of the choice of the relevant welfare ranges. Are the implications discussed here acceptable? Well, the aim of the consequentialist principle here is to give a Utilitarian framework taking care of the principle of the neutrality. One interpretation of the Repugnant Conclusion, or the two first versions of it given above, is that its repugnancy consists in its being an extreme case of sacrificing the interest of existing individuals for the sake of purely hypothetical individuals. I am not here interested in other potential reasons for its repugnancy, e.g. reasons related to the Utilitarian interpersonal aggregation of welfare per se. Also, if e.g. all individuals in *a* live above the zero level, and some individuals in *z* live below the zero level, *a* is the only permissible world, according to condition 3.
3. The unacceptable implication of e.g. consequentialist principles prescribing simply maximizing average welfare in cases like Parfit’s Two Hells (Parfit 1984, p. 392), is avoided. Assume that we have the set of worlds

**Figure 3:**
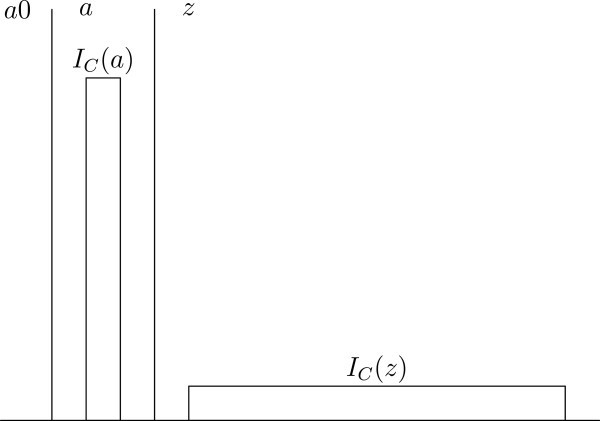
**The Repugnant Conclusion with non-overlapping populations.**
  In this case, *h* is the only permissible world, because it satisfies condition 1, and *h* + does not satisfy condition 2, because the welfare of the added people in *h* + is below zero (see Figure 4). Assume that we have the set of worlds

**Figure 4:**
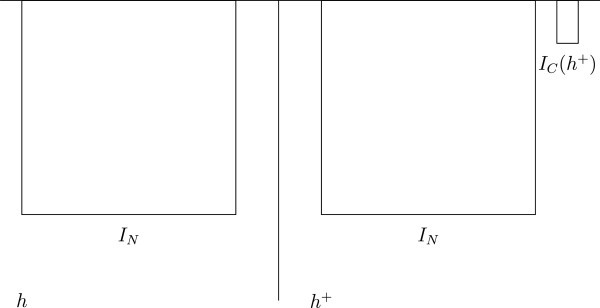
**Two Hells.**
  Still, *h* is the only permissible world, because no world satisfies any of the conditions 1–3, and *h* is the only world satisfying condition 4, because it has the highest total welfare (see Figure 5).
4. As regards Parfit’s so-called Mere Addition Paradox (Parfit 1984, p. 418–419), we note the following. Assume that we have the set of worlds

**Figure 5:**
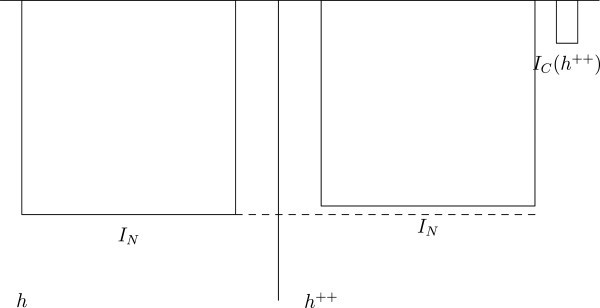
**Two Hells with a small benefit to the necessary people.**
  In this case, it would only be permissible to realize *a*, because it satisfies condition 1, and *a* + does not satisfy condition 2, because the contingent people would be better off in *b*. However, if we had the set *W*^′^ = *W*−{*b*}, it would be permissible to realize any of *a* and *a*+, because *a* satisfies condition 1, and *a* + satisfies condition 2 (see Figure 6). Thus, an axiology corresponding to (NeuW), in the sense that impermissible alternatives are always ranked lower than permissible alternatives, would violate the so-called Mere Addition Principle given the set *W*, but not given the set *W*^′^ (Arrhenius 2000, p. 60).
5. As regards Non-Identity Problems like Parfit’s 14-year old girl, where we can choose between creating a person, which would live at a level of welfare which would be low, but above the zero-point, one time and creating another person, which would live at a higher level of welfare, at another time (Parfit 1984, p. 357–363), assume we have the set of worlds

**Figure 6:**
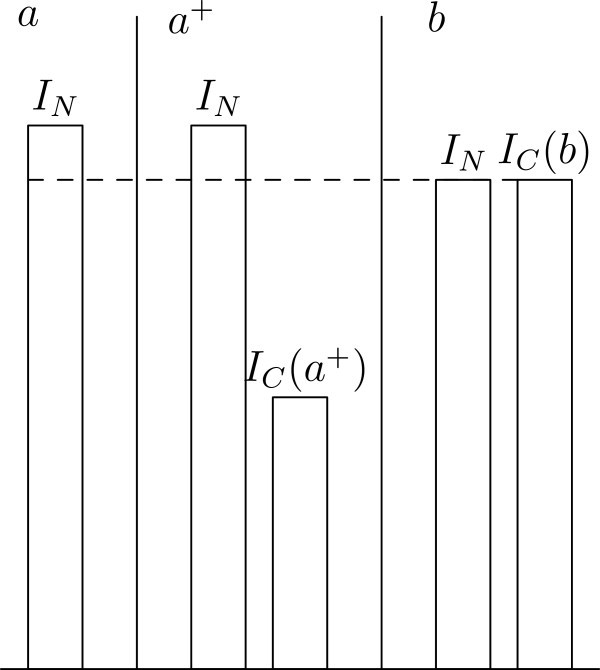
**Mere Addition.**
  In this scenario, *n*+ is the only permissible world, because it satisfies condition 2, and no world satisfies condition 1 (see Figure 7).
6. The principle has antinatalist implications of the same type as (NeuCf) discussed in the main part of this paper, because of conditions 2 and 3, which give priority to worlds where no added person lives below the zero level of welfare.

**Figure 7:**
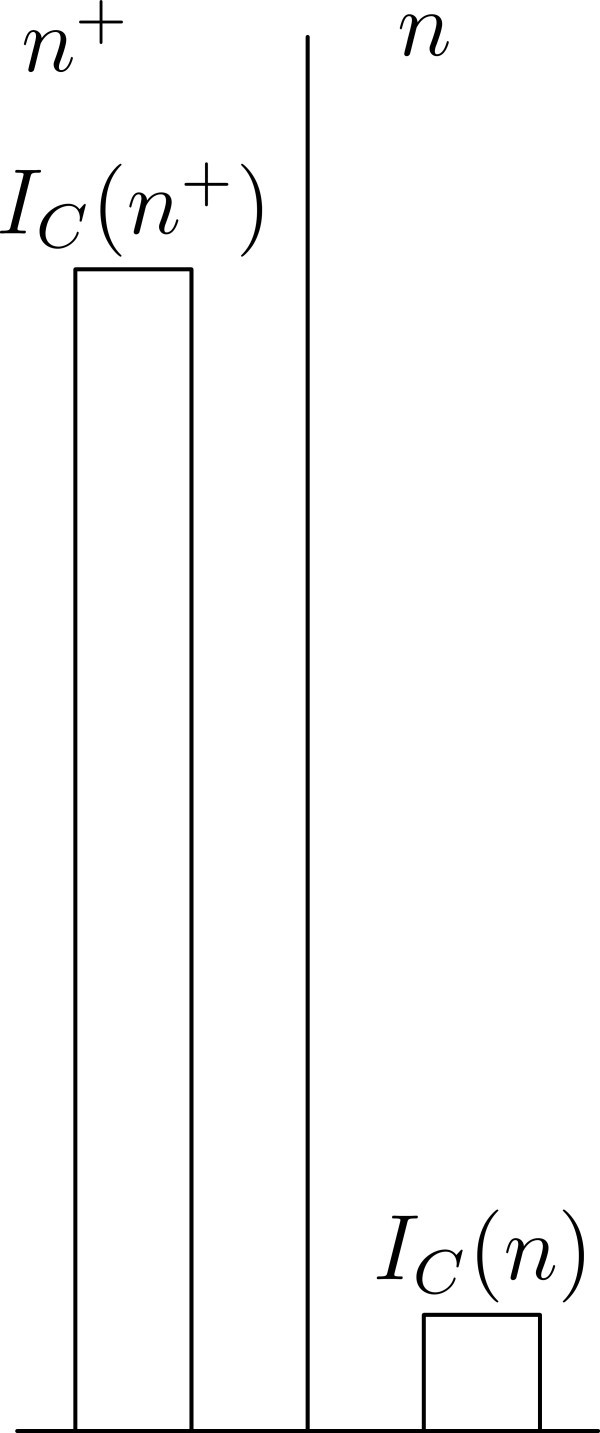
**The Non-Identity Problem.**

## Concluding remarks

I have discussed how to interpret an intuition of neutrality about future people with good lives, that may be inherent in common sense morality. I have argued that the most plausible interpretation of this intuition can avoid giving rise to antinatalist moral reasons, i.e. reasons against creating new people, in cases where we consider individual choices about having children. However, in some other situations, e.g. social policy choices affecting the identity of future people, such antinatalist implications are not so easily avoided. In order to coherently avoid supporting antinatalist policies, common sense morality has to appeal to other, outweighing moral reasons. The paper concludes with a principle of welfare promotion, which is intended to respect the most plausible interpretation of the counterfactual version of the neutrality intuition.

## Consent

Not applicable, as this paper does not refer any actual patient cases.

## Endnotes

^a^The ∀-quantifier (with ∃ defined in terms of ∀ the usual way in first-order logic) and the existence predicate could be given standard formal truth-conditions for a fixed-domain ∀-quantifier, and a variable-domain existence predicate, as in e.g. (Cresswell 2001, p. 147, 151).^b^As pointed out by one referee, things get more complicated if we allow for incommensurability in welfare. I ignore these complications here.^c^As noted by one referee, this goes against the so-called Person-Affecting Principle, according to which an outcome *o*1 only can be better than another outcome *o*1 if *o*1 is better than *o*2 for some person, and Parfit thinks a belief in this general principle, is part of common sense, so one might ask how this line of reasoning fits with the aim of this part of the paper, i.e. explicating common sense intuitions. However, Parfit argue against the Person-Affecting Principle by giving examples of particular cases, where the principle gives, according to common sense, wrong results.^d^The empirical reasoning, that the policy choice in a situation like Depletion would lead to disjoint populations in a couple of centuries, might not be commonsensical, but the moral opinion that we ought to conserve resources in a case such described still seems to be part of common sense.^e^Cf. (Lewis 2001, p. 97f). This point was stressed by one referee. However, this is realistic to assume in contexts of human agency, I think.

## Appendix

### Formalization of the intuitions and principles

#### The counterfactual interpretations

***(NeuCf)*** There is a number *a*, such that:

1. For any possible individual *x*, if *y* is an individual and *p* is a state such that Do(*y*,*p*) and ¬Do(*y*,*p*) are both possible, in a morally relevant sense, Do(*y*,*p*) *lw*(*x*) < *a* and ¬Do(*y*,*p*) ¬E(*x*), there is a moral reason for ¬Do(*y*,*p*) rather than Do(*y*,*p*).
2. For any possible individual *x*, if *y* is an individual and *p* is a state such that Do(*y*,*p*) and ¬Do(*y*,*p*) are both possible, in a morally relevant sense, Do(*y*,*p*) *lw*(*x*) ≥ *a* and ¬Do(*y*,*p*) ¬E(*x*), there is no moral reason for Do(*y*,*p*) rather than ¬Do(*y*,*p*), or vice versa, in terms of the well-being of *x*.

***(NeuCfUB)*** There is an interval [*a*,*b*], with *b* > *a*, such that:

1. For any possible individual *x*, if *y* is an individual and *p* is a state such that Do(*y*,*p*) and ¬Do(*y*,*p*) are both possible, in a morally relevant sense, Do(*y*,*p*) *lw*(*x*) < *a* and ¬Do(*y*,*p*) ¬E(*x*), there is a moral reason for ¬Do(*y*,*p*) rather than Do(*y*,*p*).
2. For any possible individual *x*, if *y* is an individual and *p* is a state such that Do(*y*,*p*) and ¬Do(*y*,*p*) are both possible, in a morally relevant sense, Do(*y*,*p*) *lw*(*x*)∈[*a*,*b*] and ¬Do(*y*,*p*) ¬E(*x*), there is no moral reason for Do(*y*,*p*) rather than ¬Do(*y*,*p*), or vice versa, in terms of the well-being of *x*.
3. For any possible individual *x*, if *y* is an individual and *p* is a state such that Do(*y*,*p*) and ¬Do(*y*,*p*) are both possible, in a morally relevant sense, Do(*y*,*p*) *lw*(*x*) > *b* and ¬Do(*y*,*p*) ¬E(*x*), there is a moral reason for Do(*y*,*p*) rather than ¬Do(*y*,*p*) (and no moral reason for ¬Do(*y*,*p*) rather than Do(*y*,*p*) in terms of the well-being of *x*).

#### Sets of individuals

Let *EF* denote the collective consisting of the couple Eve and Frank, wondering about whether to have a child.

Let *CB* denote the set of individuals “brought about” by Eve’s and Frank’s conceiving, *CB*={*x*|Do(*EF*,*c*) E(*x*)∧¬Do(*EF*,*c*) ¬E(*x*)}.

Let *NCB*={*x*|Do(*EF*,*c*) ¬E(*x*)∧¬Do(*EF*,*c*) E(*x*)}.

#### The do-interpretations

***(NeuCa)*** There is a number *a*, such that:

1. For any possible individual *x*, if *y* is an individual and Do(*y*,E(*x*)∧*lw*(*x*) < *a*) and Do(*y*,¬E(*x*)) are both possible, in a morally relevant sense, there is a moral reason for Do(*y*,¬E(*x*)) rather than Do(*y*,E(*x*)∧*lw*(*x*) < *a*).
2. For any possible individual *x*, if *y* is an individual and Do(*y*,E(*x*)∧*lw*(*x*) ≥ *a*) and Do(*y*,¬E(*x*)) are both possible, in a morally relevant sense, there is no moral reason for Do(*y*,E(*x*)∧*lw*(*x*) ≥ *a*) rather than Do(*y*,¬E(*x*)), or vice versa, in terms of the well-being of *x*.

***(NeuCaUB)*** There is an interval [*a*,*b*], with *b* > *a*, such that:

1. For any possible individual *x*, if *y* is an individual and Do(*y*,E(*x*)∧*lw*(*x*) < *a*) and Do(*y*,¬E(*x*)) are both possible, in a morally relevant sense, there is a moral reason for Do(*y*,¬E(*x*)) rather than Do(*y*,E(*x*)∧*lw*(*x*) < *a*).
2. For any possible individual *x*, if *y* is an individual and Do(*y*,E(*x*)∧*lw*(*x*)∈[*a*,*b*]) and Do(*y*,¬E(*x*)) are both possible, in a morally relevant sense, there is no moral reason for Do(*y*,E(*x*)∧*lw*(*x*)∈[*a*,*b*]) rather than Do(*y*,¬E(*x*)), or vice versa, in terms of the well-being of *x*.
3. For any possible individual *x*, if *y* is an individual and Do(*y*,E(*x*)∧*lw*(*x*) > *b*) and Do(*y*,¬E(*x*)) are both possible, in a morally relevant sense, there is a moral reason for Do(*y*,E(*x*)∧*lw*(*x*) > *b*) rather than Do(*y*,¬E(*x*)) (and there is no moral reason for Do(*y*,¬E(*x*)) rather than Do(*y*,E(*x*)∧*lw*(*x*) > *b*) in terms of the well-being of *x*).

#### The principle (NeuW)

***(NeuW)*** Assume that we have a set of worlds *W*, a set of necessary individuals *I*_*N*_ = {*x*|∀*w*∈*W*⊧_*w*_E(*x*)}. *I*_*C*_(*w*) yields the set of contingent individuals relative to a world *w*, *I*_*C*_(*w*) = {*x*|*x*∉*I*_*N*_∧⊧_*w*_E(*x*)}. Let *tw*(*I*) denote the total welfare of the individuals in set *I*. If *I* = *∅*, let *tw*(*I*) = 0. It is permissible to realize a world *w* out of *W* iff *w* satisfies the following disjunctive condition with respect to any *W*^′^⊆*W* such that *w*∈*W*^′^:

1. The total welfare *tw*(*I*_*N*_) of the necessary people in *w* is at least as high as the *tw*(*I*_*N*_) in any other *w*^′^∈*W*^′^, and *I*_*C*_(*w*) = *∅*, OR
2. *tw*(*I*_*N*_) in *w* is at least as high as *tw*(*I*_*N*_) in any other *w*^′^∈*W*^′^, and *tw*(*I*_*C*_(*w*)) in *w* is at least as high as *tw*(*I*_*C*_(*w*^′^)) in *W*^′^, for any other world *w*^′^∈*W*^′^, and *tw*({*x*}) ≥ 0 for any individual *x*∈*I*_*C*_(*w*) OR
3. if there is no world satisfying any of the above conditions, and if there is a non-empty set *W*^′′^⊂*W*^′^, such that *W*^′′^ = {*w*∈*W*|∀*x*∈*I*_*C*_(*w*)(*w*⊧*tw*({*x*}) ≥ 0)}, *w*∈*W*^′′^ and *tw*(*I*_*N*_∪*I*_*C*_(*w*)) in *w* is at least as high as *tw*(*I*_*N*_∪*I*_*C*_(*w*^′^)) in *W*^′^, for any other world *w*^′^∈*W*^′′^ OR
4. if there is no world satisfying any of the above conditions, *tw*(*I*_*N*_∪*I*_*C*_(*w*)) in *w* is at least as high as *tw*(*I*_*N*_∪*I*_*C*_(*w*^′^)) in *W*^′^, for any other world *w*^′^∈*W*^′^.
